# Expression of P2 receptors in human B cells and Epstein-Barr virus-transformed lymphoblastoid cell lines

**DOI:** 10.1186/1471-2172-7-22

**Published:** 2006-09-14

**Authors:** Dong Hyeon Lee, Kyu Sang Park, In Deok Kong, Jun Woo Kim, Bok Ghee Han

**Affiliations:** 1 Biobank for Health Sciences, Center for Genome Sciences, National Institute of Health, Korea Center for Disease Control and Prevention, Seoul, South Korea; 2 Department of Physiology, Institute of Basic Medical Science, Yonsei University, Wonju College of Medicine, Wonju, South Korea

## Abstract

**Background:**

Epstein-Barr virus (EBV) infection immortalizes primary B cells in vitro and generates lymphoblastoid cell lines (LCLs), which are used for several purposes in immunological and genetic studies. Purinergic receptors, consisting of P2X and P2Y, are activated by extracellular nucleotides in most tissues and exert various physiological effects. In B cells, especially EBV-induced LCLs, their expression and function have not been well studied. We investigated the expression of P2 receptors on primary human B cells and LCLs using the quantitative reverse transcriptase-polymerase chain reaction (RT-PCR) method for revealing the gene expression profile of the P2 receptor subtypes and their changes during transformation.

**Results:**

The mRNA transcripts of most P2 receptors were detected in primary B cells; the expression of P2X_3 _and P2X_7 _receptors was the lowest of all the P2 receptors. By contrast, LCLs expressed several dominant P2 receptors – P2X_4_, P2X_5_, and P2Y_11 _– in amounts similar to those seen in B cells infected with EBV for 2 weeks. The amount of most P2 subtypes in LCLs or EBV-infected B cells was lower than in normal B cells. However, the amount of P2X_7 _receptor expressed in LCLs was higher. Protein expression was studied using Western blotting to confirm the mRNA findings for P2X_1_, P2X_4_, P2X_7_, P2Y_1_, and P2Y_11 _receptors. ATP increased the intracellular free Ca^2+ ^concentration ([Ca^2+^]_i_) by enhancing the Ca^2+ ^influx in both B cells and LCLs in a dose-dependent manner.

**Conclusion:**

These findings describe P2 receptor expression profiles and the effects of purinergic stimuli on B cells and suggest some plasticity in the expression of the P2 receptor phenotype. This may help explain the nature and effect of P2 receptors on B cells and their role in altering the characteristics of LCLs.

## Background

B cells synthesize and secrete large quantities of soluble immunoglobulin antibodies and thus, play a key role in humoral immunity. An infection with the Epstein-Barr virus (EBV) easily transforms resting primary B cells in vitro from human peripheral blood cells into B-blast-like proliferating lymphoblastoid cell lines (LCLs) [1]. This infection is used routinely in the laboratory to generate LCLs from B cells [2]. LCLs are widely used in various types of studies, including those involving the disciplines of immunology, cellular biology, and genetics. This transformation results in changes in certain cellular properties, including gene expression [3], cell surface phenotyping, and cytokine production [4].

Extracellular nucleotides – e.g., adenosine 5'-triphosphate (ATP), adenosine 5'-diphosphate, uracil 5'-triphosphate, and uracil 5'-diphosphate – have various physiological effects in many cells, such as exocrine and endocrine secretion, neurotransmission, cell proliferation, cell differentiation, and programmed cell death that are mediated by P2 receptors, consisting of P2X and P2Y receptors [5]. P2X receptors are ligand-gated cation channels, of which seven receptor subtypes (P2X_1 _to P2X_7_) have been identified and cloned [6]. P2Y receptors, which are G-protein-coupled metabotropic structures, consist of eight cloned and functionally distinct subtypes: P2Y_1_, P2Y_2_, P2Y_4_, P2Y_6_, P2Y_11_, P2Y_12_, P2Y_13_, and P2Y_14 _[5,7].

Blood cells express P2 receptors which regulate such responses as cell proliferation, differentiation, chemotaxis, cytokine release, immune and inflammatory responses [5,8]. In lymphocytes, ATP induces an increase in membrane permeability for cations and larger molecules [9,10], as well as cellular proliferation [11] and cell death through P2 receptors [12,13]. The precise nature of the expression and function of the P2 receptor subtypes have been investigated [14-16].

P2 receptors expressed in B cells have been investigated using electrophysiological, pharmacological, and immunocytochemical techniques, which have revealed the existence of P2 receptors [17], especially P2X [14,18]. However, the researchers in these studies failed to perform a quantitative analysis of P2 mRNA and used B cells from chronic lymphocytic leukemia (CLL) or LCLs, rather than pure B cells. Recently, the mRNA profile of the lymphocyte P2 receptor was subjected to quantitative analysis, but the B cells were not separated and not all subtypes were targeted [15,16].

In this study, we investigated the expression of P2 receptors in human B cells and in LCLs using quantitative reverse transcriptase-polymerase chain reaction (RT-PCR), Western blotting, and fluorimetric techniques to measure intracellular free Ca^2+ ^concentration ([Ca^2+^]_i_). We were able to determine the profile of the P2 receptor mRNA in these cells and monitor changes in [Ca^2+^]_i _in response to P2 receptor activation. Our findings indicate the plasticity of P2 receptors in B cells during their transformation into LCLs.

## Results

IgD and CD38 are cell-surface molecules that have been used widely to identify the B-cell phenotype during B-cell development. Like germinal center B cells, most EBV-transformed B cells were positive for CD38 but not for IgD [19,20]. The expression of IgD and CD38 molecules on primary B cells and EBV-transformed LCLs was evaluated by fluorescence-activated cell sorter (FACS) analysis. To generate LCLs, we cultured isolated B cells with the active EBV supernatant for 4 to 6 weeks, as described in *Methods *section. The primary B cells expressed IgD, but not CD38, and the LCLs expressed CD38, but not IgD (data not shown). This result is consistent with our previous findings [20].

### P2 receptor mRNA quantification

**Figure 1:**
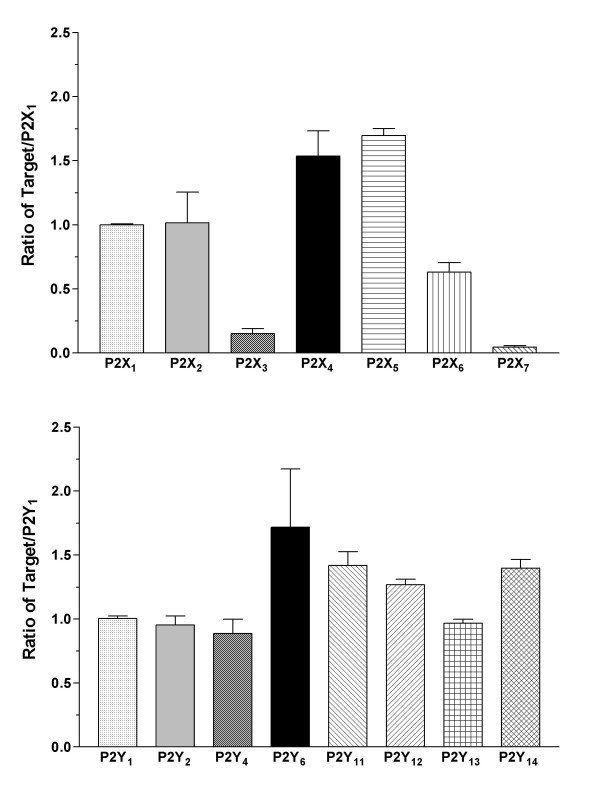
**Relative expression of P2 receptors in B cells**. The relative expression of the P2X receptor gene (upper; P2X_1_, P2X_2_, P2X_3_, P2X_4_, P2X_5_, P2X_6_, and P2X_7_) and P2Y receptor gene (lower; P2Y_1_, P2Y_2_, P2Y_4_, P2Y_6_, P2Y_11_, P2Y_12_, P2Y_13_, and P2Y_14_) in B cells are presented (n = 4). The expression was normalized to GAPDH. P2X receptors were calibrated by P2X_1 _and P2Y receptors were calibrated by P2Y_1_. Data are the mean ± SEM.

**Figure 2:**
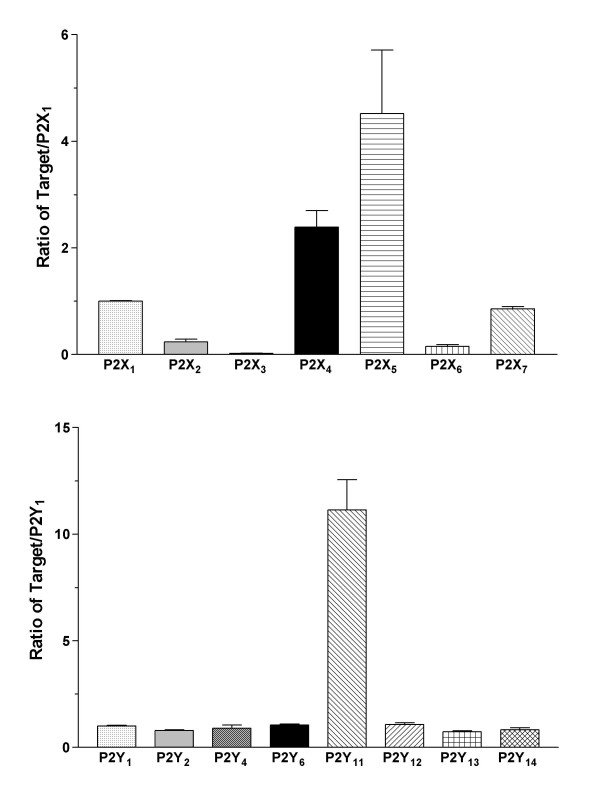
**Relative expression of P2 receptors in LCLs**. The relative expression of the P2X receptor gene (upper) and P2Y receptor gene (lower) in EBV-infected B cells (2 wks), LCLs, are presented (n = 4). The expression was normalized to GAPDH. P2X receptors were calibrated by P2X_1 _and P2Y receptors were calibrated by P2Y_1_. Data are the mean ± SEM.

**Figure 3:**
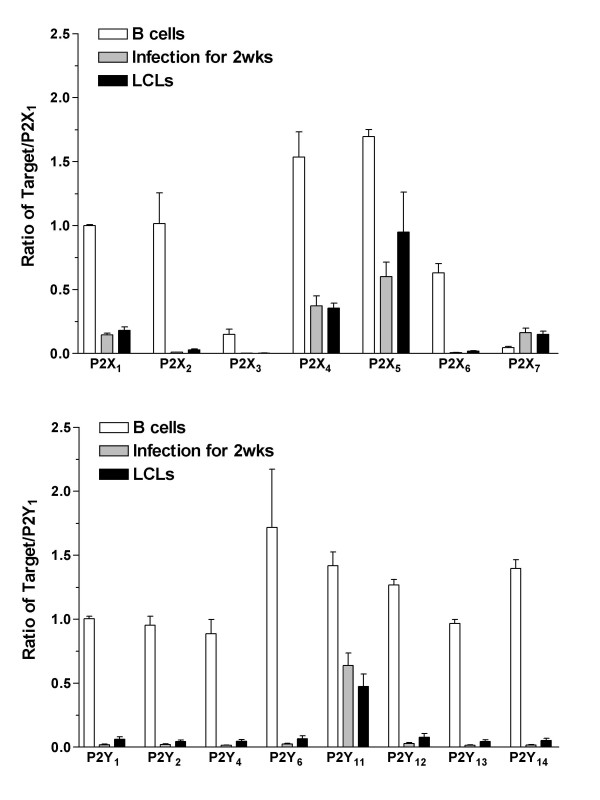
**Comparison of relative P2 receptor expression in B cells and LCLs**. The relative expression of the P2X receptor gene (upper) and P2Y receptor gene (lower) in B cells, EBV-infected B cells for 2 weeks, and LCLs are presented (n = 4). The expression was normalized to GAPDH. P2X receptors were calibrated by P2X_1 _of B cells and P2Y receptors were calibrated by P2Y_1 _of B cells. Data are the mean ± SEM.

**Figure 4:**
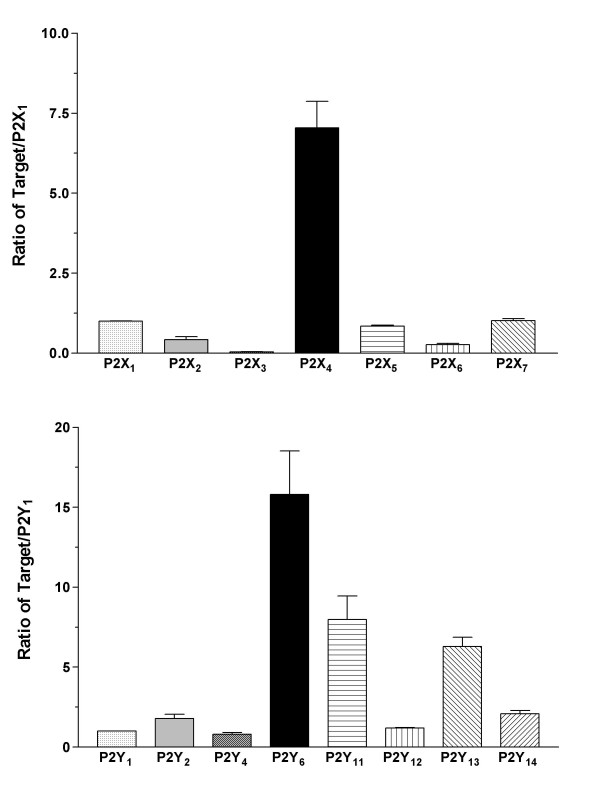
**Relative expression of P2 receptors in PBMCs**. The relative expression of the P2X receptor gene (upper) and P2Y receptor gene (lower) in PBMCs are presented (n = 4). The expression was normalized to GAPDH. P2X receptors were calibrated by P2X_1 _and P2Y receptors were calibrated by P2Y_1_. Data are the mean ± SEM.

The expression of P2 receptors in B cells and LCLs was determined using quantitative RT-PCR. The expression of the P2 receptor subtypes was compared among B cells, LCLs, and peripheral blood mononuclear cells (PBMCs). P2X_1 _or P2Y_1 _were used as a calibrator (i.e. the P2X receptor was expressed as a ratio of P2X_1 _and the P2Y receptor as P2Y_1_) in order to illustrate the expression of P2 receptors relative to each other. All P2X and P2Y receptor subtypes were detected in the B cells. Most of the P2 receptor subtypes had similar rates of expression within 1- or 2-fold of each other with the exception of the P2X_3 _and P2X_7 _receptors, which were expressed in lower quantities (Figure 1, *n *= 4). P2X_7 _receptor expression was significantly low compared to other P2X receptors (*p *< 0.05), with the exception of P2X_3_. The presence of P2X- and P2Y-receptor mRNA in the B cells is in agreement with the findings of previous lymphocyte studies using RT-PCR [15,16]. EBV-infected B cells were also examined because an in vitro transformation might alter the expression of receptors. The most abundant P2 receptor subtypes were P2X_4_, P2X_5_, and P2Y_11 _(Figure 2; *n *= 4). The expression of P2X_5 _receptors in LCLs was significantly higher than other P2X receptors (*p *< 0.05; Figure 2). The P2 receptors in B cells were compared with those expressed in LCLs (Figure 3, P2X_1 _of B cells used as a P2X calibrator and P2Y_1 _as a P2Y calibrator). The expression of the P2X_1 _through to P2X_6 _receptors and P2Y receptors in LCLs and B cells that had been infected with EBV for 2 weeks was significantly lower than in noninfected B cells (*p *< 0.01; Figure 3). However, the LCLs expressed a significantly larger number of P2X_7 _receptors than B cells (*p *< 0.01; Figure 3). The expression of EBV-infected LCLs, which had been infected for more than 4 weeks and EBV-infected B cells, which had been infected for 2 weeks, yielded similar profiles and quantities. As a control, P2 receptors in PBMCs were quantified and these showed a different expression profile. In PBMCs, which are mainly monocytes and lymphocytes, P2X_4_, P2Y_6_, P2Y_11_, and P2Y_13 _were the predominant P2 receptor subtypes (Figure 4, *n *= 4), and the expression rates for P2X_4 _and P2Y_6 _were significantly higher than other P2X or P2Y subtypes (*p *< 0.001). In addition, P2X_4_, P2X_7_, P2Y_6_, P2Y_11_, and P2Y_13 _expression was significantly higher in PBMCs compared with B cells (*p *< 0.05), which may be the result of T cell/monocyte contamination in the PBMC preparation [15,16]. Therefore, the up-regulated P2X_7 _receptor can be expected to have a physiological role during the transformation of B cells into LCLs.

### Western blotting for P2 receptors

**Figure 5:**
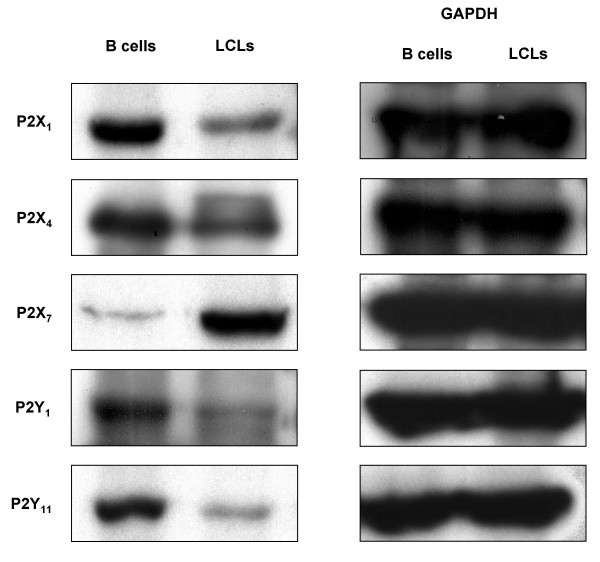
**P2 receptors (P2X_1_, P2X_4_, P2X_7_, P2Y_1_, and P2Y_11_) compared by Western blotting in B cells and LCLs**. Proteins extracted from B cells and LCLs were probed with rabbit polyclonal antibodies directed against P2X_1 _(60-kDa), P2X_4 _(65-kDa), P2X_7 _(68-kDa), P2Y_1 _(66-kDa), and P2Y_11 _(50-kDa) (left). Membranes were re-probed by GAPDH (40-kDa) (right). Data are representatives of 4 independent experiments.

To investigate the correlation of mRNA with protein, we carried out Western blot analysis for P2X_1_, P2X_4_, P2X_7_, P2Y_1_, and P2Y_11 _receptors, all of which had varying amounts of mRNA during EBV transformation (*n *= 4). The distribution of P2 receptors in B cells and LCLs is shown on the left panel (Figure 5). The bands representing P2X_1 _(60-kDa), P2X_4 _(65-kDa), P2Y_1 _(66-kDa), and P2Y_11 _(50-kDa) receptors were more prominent in B cells than in LCLs, which correlates with the results of the mRNA quantitative analysis. As for the P2X_7 _receptor, it was represented by a prominent 68-kDa band in LCLs and a faint band in B cells. This is consistent with the results of RT-PCR, which indicated that the expression of P2X_7 _is higher in LCLs than in B cells. To compare protein loading, the blot was re-probed with anti-glyceraldehyde-3-phosphate dehydrogenase (GAPDH) antibody (40-kDa) (Figure 5, right).

### Effect of ATP on intracellular free Ca^2+ ^concentration

**Figure 6:**
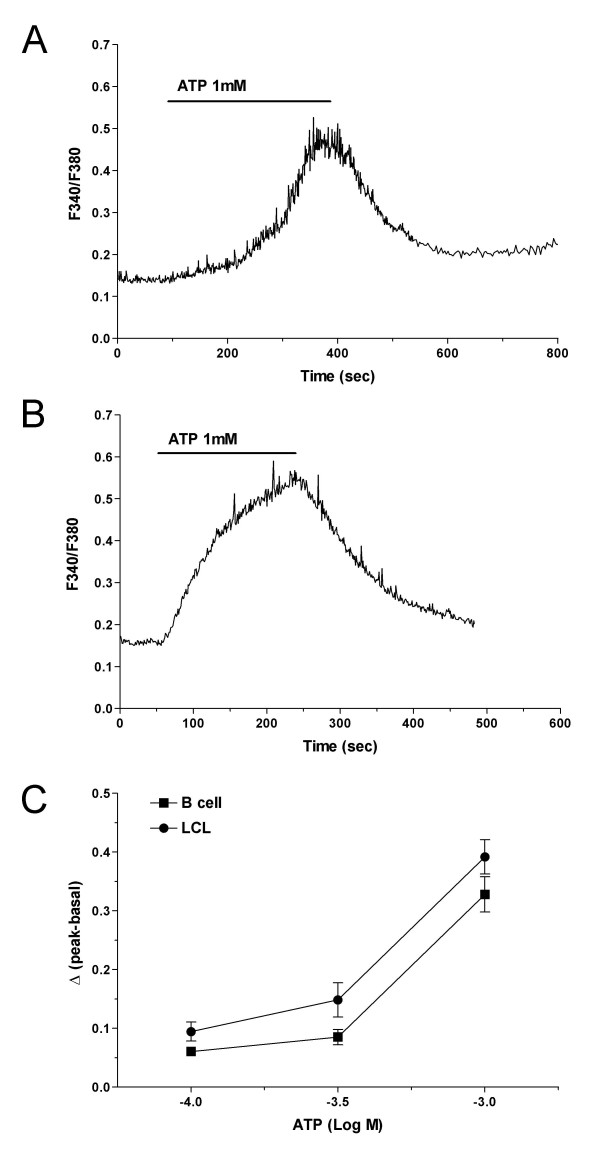
**Comparison of ATP-induced increases in [Ca^2+^]_i _in B cells and LCLs**. (A, B) Average time courses for Ca^2+ ^responses to P2 receptor stimulation with ATP (1 mM) in B cells (A, *n *= 6) and in LCLs (B, *n *= 6). ATP was applied as indicated by the horizontal bars above the traces. (C) Concentration-response curve for the increase in [Ca^2+^]_i _in response to ATP in B cells and in LCLs (Δratio F340/F380). ATP increased [Ca^2+^]_i _in a dose-dependent manner. The maximum increase in the [Ca^2+^]_i _over basal is plotted against the concentration of ATP. The measurement of [Ca^2+^]_i _was carried out in single cells attached to a coverslip, as described in the *Methods *section. Data are the mean ± SEM for at least 25 cells on 4 to 5 coverslips.

**Figure 7:**
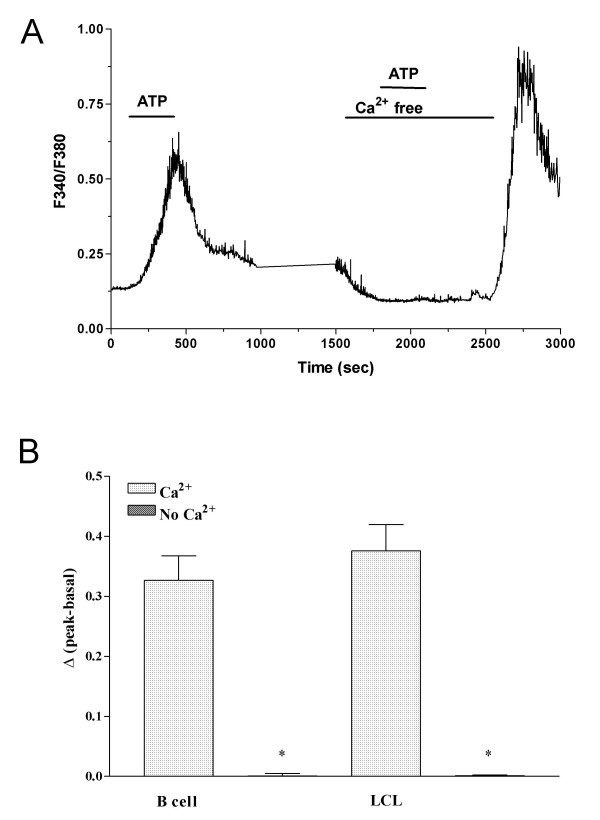
**Effect of extracellular Ca^2+^-free conditions on [Ca^2+^]_i _induced by ATP**. (A) Average time courses for Ca^2+ ^responses induced by external perfusion of ATP (1 mM) in normal solution and the Ca^2+^-free solution in B cells (*n *= 4). Agent was applied as indicated by the horizontal bars above the traces. (B) Peak of ATP-induced [Ca^2+^]_i _rises during pretreatment with the Ca^2+^-free solution (*n *= 14 for B cells; *n *= 12 for LCLs). Data are the mean ± SEM; *p < 0.0001 compared to Ca^2+ ^treated samples.

Extracellular ATP is an effective modulator of [Ca^2+^]_i_, and its activities are mediated through P2 receptors [17]. To determine whether the P2 receptors examined by RT-PCR and Western blotting were functional, we carried out digital fluorescence imaging of the Ca^2+^-sensitive dye Fura 2-AM and compared the images seen following ATP-induced changes in [Ca^2+^]_i _in B cells and LCLs. In the presence of 2 mM Ca^2+ ^and 1 mM ATP, we observed an average increase in [Ca^2+^]_i _from 0.1 to 0.5 (representing the F340:F380 ratio) in B cells and LCLs (Δratio of B cells: 0.32 ± 0.04, *n *= 6; Δratio of LCLs: 0.39 ± 0.04, *n *= 6) (Figures 6A and 6B). The [Ca^2+^]_i _increased with ATP in a dose-dependent manner (100–1000 μM; *n *= 25) (Figure 6C), indicating that the potency of ATP was similar in both cells. The mechanism leading to the intracellular Ca^2+ ^response was examined further by repeating these experiments under Ca^2+^-free conditions. The B cells were treated with 1 mM ATP under Ca^2+^-free conditions, and the [Ca^2+^]_i _remained at or near the pre-agonist levels (Figure 7A). The peak [Ca^2+^]_i _was mostly abolished in B cells (*n *= 14, *p *< 0.0001) and in LCLs (*n *= 12, *p *< 0.0001) in the absence of Ca^2+ ^(Figure 7B). These data suggest that an influx of Ca^2+ ^is the major route by which B cells and LCLs respond to ATP stimulation.

## Discussion

In this study, we determined and compared mRNA expression levels for all known P2X and P2Y receptor subtypes on human B cells and LCLs. Quantitative RT-PCR was used to determine the gene expression profile for P2 receptors. This method was selected because selective agonists and antagonists for most of the P2 receptor subtypes are absent and real-time PCR has advantages over other methods, such as requiring only a small number of cells and being one of the most reliable methods of determining the amount of RNA.

This is the first study to show the expression of P2 receptors using mRNA from healthy human B cells. In these cells, most of the P2X and P2Y receptor subtypes had 2-fold expression with the exception of P2X_3 _and P2X_7 _receptors. In the studies of the P2X receptor, the P2X_1_, P2X_2_, P2X_4_, and P2X_7 _receptors were found in human B cells by an immunocytochemical assay [14] and the non-desensitizing cation channels activated by ATP, which is a feature of P2X_7 _receptor, were measured using electrophysiological methods [18]. The different results of P2X subtype expression might be due to the different B cells, or variations in P2X receptor expression [21]. B cells transformed by EBV [18] or malignant B cells [14] were used in previous studies, while normal B cells were used in the present study. In addition, it is possible that there might be differences in the transcription, translation, and function of P2X receptors. The different P2X_7 _expression levels may be because P2X_7 _receptor might be up-regulated in CLLs [10] and that some lymphoid cells do not express P2X_7 _receptor [11]. In addition, B cells did not undergo the typical increase in membrane permeability to ATP and were not susceptible to ATP-mediated cytotoxicity [8,22]. Although the P2Y receptors in B cells were investigated, it was not enough to compare the expression of subtypes. P2Y subtypes were detected by RT-PCR in previous studies, albeit only in lymphocytes [15,16].

In LCLs and B cells infected by EBV for 2 weeks, the predominant P2 receptor subtypes were P2X_1_, P2X_4_, P2X_5_, P2X_7_, and P2Y_11_. The expression of most P2 receptors was suppressed during the EBV-induced B-cell transformation into LCLs, however, the suppression of P2X_1_, P2X_4_, P2X_5 _and P2Y_11 _receptors was not as great as for other subtypes. Only P2X_7 _receptor was significantly up-regulated. Western blotting showed similar patterns for P2X_1_, P2X_4_, P2X_7_, P2Y_1_, and P2Y_11_, as well as for P2X_2_, P2X_5_, P2Y_2_, and P2Y_6 _(data not shown). Our results suggest that there is some plasticity in P2-receptor expression in B cells. This possibility has been investigated in many tissues and cells, including the urinary bladder, heart, vessels, gut, neurons, and cancer cells [5]. In immune cells, plasticity in P2Y_2_-receptor expression was studied during myeloid leukocyte differentiation [23]. Sensitivity to ATP in thymocytes changes with the stage of maturation [24,25], and P2X_7_-receptor expression can be modulated by diverse stimuli [26]. The plasticity of P2 receptors may be due to changes in their exposure to ATP or EBV-induced changes in gene expression. In vivo, ATP is often released by blood cells into the extracellular environment through nonlytic mechanisms. Some leakage of cytoplasmic ATP may also occur as a consequence of damage to the cell or acute cell death. Platelet-dense granules comprise another relevant source of ATP [8]. In vitro, however, the sources of ATP for B cells are limited to nonlytic mechanisms or leakage of cytoplasmic ATP. The EBV-induced transformation of B cells into LCLs results in some B cells dying, which results in ATP being released into the extracellular compartment, where it continually degrades. Thus, the concentration of ATP may be high in the early stages of in vitro transformation and lower in later stages. The expression of P2 receptors may be affected by this fluctuation in environmental ATP.

In PBMC populations that include lymphocytes and monocytes, the dominant P2 receptor subtypes were P2X_4_, P2Y_6_, P2Y_11_, and P2Y_13_. An mRNA expression assay revealed that the P2Y_1_, P2Y_2_, P2Y_4_, and P2Y_6 _receptors were expressed in lymphocytes and monocytes and that the P2Y_6 _receptor was expressed in relatively higher amounts than the other P2Y receptor subtypes [16]. P2X_4 _and P2Y_12 _receptors were expressed in relatively large amounts in lymphocytes and P2X_4_, P2Y_2_, and P2Y_13 _receptors in monocytes [15]. The expression of P2X_4_, P2Y_6_, and P2Y_13 _receptors correlated with the findings of previous studies; however, the expression of the P2Y_11 _receptor was somewhat different. It is possible that other lymphocytes or monocytes expressed these subtypes predominantly. Alternatively this may reflect a variation in cohorts or contamination with other types of blood cells. To date, these blood cells have not been investigated well enough to compare P2 receptor subtypes, although some of them have been surveyed [5,8,16,27,28]. Because the P2 receptor profiles of blood cells are not completely known, it is difficult to determine which P2 receptor subtypes have been expressed dominantly in PBMCs until now.

Although the P2Y_8 _and P2Y_10 _receptors were examined with other subtypes, the findings for these subtypes were omitted because they are not included among the classical P2Y receptor subtypes in humans. We found the mRNA for these subtypes in B cells, LCLs, and PBMCs, indicating that they are prominent in these cells. In previous studies of human P2 receptors, the P2Y_8 _and P2Y_10 _receptors were expressed in HL60 [29] and included in the human genome [30]. The National Center for Biotechnology Information (NCBI) confirmed the gene sequence for each of these receptors (P2Y_8_; NM_178129, P2Y_10_; NM_014499). A physiological role for the P2Y_8 _and P2Y_10 _receptors in B cells and in human blood cells can therefore be expected.

ATP-stimulated P2 receptors increased the [Ca^2+^]_i _in B cells and LCLs, albeit rather slowly. This was quite a different effect from that of other stimuli, such as the anti-IgM antibody, which caused [Ca^2+^]_i _levels to change rapidly [31]. This might be due to differences in Ca^2+ ^signaling or the temperature at which the experiments were conducted, which might influence the kinetics involved when the [Ca^2+^]_i _changes. Extracellular Ca^2+^-free conditions prevented the [Ca^2+^]_i _from increasing, thereby indicating that the main cause of the increase in [Ca^2+^]_i _might be an ATP-induced influx of Ca^2+^, although the possibility that mobilization of stored Ca^2+ ^may be involved should probably also be considered. The increased [Ca^2+^]_i _might be largely due to P2X receptor activity, because it was mediated by an influx of Ca^2+^, which is the major effect of activating P2X receptors. The results of real-time PCR indicated a decrease in P2 receptors and those of Western blotting demonstrated a similar pattern for several P2 receptors, even when the up-regulation of P2X_7 _receptor was considered. However, the increase in [Ca^2+^]_i _by ATP was a little higher in LCLs than in B cells, which was not statistically significant. EBV-transformed B cells might enhance the availability of Ca^2+^, thereby causing [Ca^2+^]_i _to rise, even when P2 receptors are down-regulated. The response to extracellular ATP is an increase in [Ca^2+^]_i _in B cells as well as in LCLs, which are probably derived from EBV-infected B cell lymphoma in vivo. This may cause a variety of cellular events, ranging from transcriptional regulation to cell migration and proliferation.

## Conclusion

In this study, the expression of P2X and P2Y receptors in human B cells and LCLs was investigated. P2-receptor expression was suppressed during the EBV-induced transformation of B cells, except for the P2X_7 _subtype, which was up-regulated. Extracellular ATP induced an increase in [Ca^2+^]_i _in B cells and LCLs via P2 receptors. Therefore, these findings reveal the exact P2 receptor profiles and the effects of purinergic stimuli on B cells and suggest some plasticity in the expression of the P2 receptor phenotype. This will help us explain the nature and effect of P2 receptors on B cells and their role in altering the characteristics of LCLs.

## Methods

### B-cell purification and generation of EBV-transformed LCLs

Ten 240-mL packs of blood were obtained from the Central Red Cross Blood Center (Seoul, Korea). This blood was not appropriate for transfusion because of slightly elevated alanine aminotransferase levels. We used it to isolate PBMCs, using Ficoll-Hypaque gradient centrifugation (Amersham Biosciences, Uppsala, Sweden) and B cells, which were purified (>95% CD20^+^) using a B-cell isolation kit and a MACS separator (Miltenyi Biotec, Bergisch Gladbach, Germany). The immortalization of B cells was achieved by EBV infection [2,32-34]. The B95-8 supernatant was added to the purified B cells in a culture flask (1 × 10^6 ^cells/mL). Following a 2-hour incubation period at 37°C, the same volume of medium and 0.5 μg/mL cyclosporine A [35] were added. The cultures were incubated for 4 to 6 weeks until clumps of EBV-infected B cells were visible. EBV-transformed LCLs were cultured in RPMI-1640 medium (GIBCO/BRL, Grand Island, NY, USA) supplemented with 10% heat-inactivated fetal bovine serum (FBS) (BioWhittaker, Walkerville, MD, USA) and 1% (v/v) antibiotics/antimycotics that included penicillin G (100 IU/mL), streptomycin (100 μg/mL), and amphotericin B (0.25 μg/mL). The cells were cultured in a humidified atmosphere of 5% CO_2 _and 95% air at 37°C. The EBV stock was prepared from an EBV-transformed B95-8 marmoset cell line. These cells were grown in an RPMI-1640 medium supplemented with 10% FBS, and infectious culture supernatants were harvested and stored at -80°C until needed. Thus, each pack of blood was used to produce B cells, EBV-infected B cells, LCLs, and PBMCs for use in this experiment. The study was approved by the Institutional Review Board at the National Institute of Health, Korea Center for Disease Control and Prevention.

### Quantitative real-time RT-PCR

The total cellular RNA was collected from human B cells, LCLs, and PBMCs. RNA was extracted using the RNeasy mini kit (Qiagen, Valencia, CA, USA) according to the manufacturer's instructions and stored at -80°C until used. Quantitative RT-PCR was performed to determine the expression of the P2 receptor genes (Table 1). To generate cDNA, we induced reverse transcription of the total RNA using oligodT_15 _(Roche Diagnostics GmbH, Mannheim, Germany) and reverse transcription polymerase (Promega, Madison, WI, USA). Oligonucleotide primers (Bioneer, Daejeon, Korea) were designed using Primer Express software (Applied Biosystems, Foster City, CA, USA), based on sequences obtained from the GenBank database, and tested for quality and efficiency. Primer efficiency was established to ensure optimal amplification of our samples. Serial dilutions of synthetic cDNAs were carried out according to the supplier's instructions to define relative changes in quantity. Real-time PCR was performed using SYBR Green PCR Master mix (Applied Biosystems) in an ABI PRISM 7900 HT Sequence Detection System (Applied Biosystems). The amplification program included activation of AmpliTaq Gold at 95°C for 10 minutes, followed by 45 cycles of 2-step PCR with denaturation at 95°C for 15 seconds and annealing/extension at 60°C for 1 minute. Amplifications were followed by a melting curve analysis. A negative control (no cDNA template) was run simultaneously with every assay. The PCR from each cDNA sample was run in triplicate. Constitutively expressed GAPDH was selected as an endogenous control to correct any potential variation in RNA loading or in the efficiency of the amplification reaction. Results are presented as relative fold changes by using GAPDH as a reference and P2X_1 _or P2Y_1 _as a calibrator and applying the formula 2^-ΔΔ*Ct *^[36].

**Table 1:** Sequence details for all P2X and P2Y receptor subtypes and reference (GAPDH) primers.

Receptor/Gene accession	Direction	Sequence	Position
P2X_1_	Forward	5'-CGCCTTCCTCTTCGAGTATGA-3'	471 – 491
NM_002558	Reverse	5'-AGATAACGCCCACCTTCTTATTACG-3'	538 – 514
P2X_2_	Forward	5'-GCCTACGGGATCCGCATT-3'	958 – 975
NM_170682	Reverse	5'-TGGTGGGAATCAGGCTGAAC-3'	1024 – 1005
P2X_3_	Forward	5'-GCTGGACCATCGGGATCA-3'	135 – 152
NM_002559	Reverse	5'-GAAAACCCACCCTACAAAGTAGGA-3'	205 – 182
P2X_4_	Forward	5'-CCTCTGCTTGCCCAGGTACTC-3'	1108 – 1128
NM_002560	Reverse	5'-CCAGGAGATACGTTGTGCTCAA-3'	1176 – 1155
P2X_5_	Forward	5'-CTGCCTGTCGCTGTTCGA-3'	311 – 328
NM_002561	Reverse	5'-GCAGGCCCACCTTCTTGTT-3'	378 – 360
P2X_6_	Forward	5'-AGGCCAGTGTGTGGTGTTCA-3'	488 – 507
AF065385	Reverse	5'-TCTCCACTGGGCACCAACTC-3'	555 – 536
P2X_7_	Forward	5'-TCTTCGTGATGACAAACTTTCTCAA-3'	401 – 425
NM_002562	Reverse	5'-GTCCTGCGGGTGGGATACT-3'	476 – 458
P2Y_1_	Forward	5'-CGTGCTGGTGTGGCTCATT-3'	1352 – 1370
NM_002563	Reverse	5'-GGACCCCGGTACCTGAGTAGA-3'	1419 – 1399
P2Y_2_	Forward	5'-GAACTGACATGCAGAGGATAGAAGAT-3'	1495 – 1520
NM_176072	Reverse	5'-GCCGGCGTGGACTCTGT-3'	1567 – 1551
P2Y_4_	Forward	5'-CCGTCCTGTGCCATGACA-3'	725 – 742
NM_002565	Reverse	5'-TGACCGCCGAGCTGAAGT-3'	793 – 776
P2Y_6_	Forward	5'-GCCGGCGACCACATGA-3'	1171 – 1186
NM_176797	Reverse	5'-GACCCTGCCTCTGCCATTT-3'	1227 – 1209
P2Y_11_	Forward	5'-CTGGAGCGCTTCCTCTTCAC-3'	511 – 530
NM_002566	Reverse	5'-GGTAGCGGTTGAGGCTGATG-3'	586 – 567
P2Y_12_	Forward	5'-AGGTCCTCTTCCCACTGCTCTA-3'	318 – 339
NM_022788	Reverse	5'-CATCGCCAGGCCATTTGT-3'	385 – 368
P2Y_13_	Forward	5'-GAGACACTCGGATAGTACAGCTGGTA-3'	223 – 248
NM_023914	Reverse	5'-GCAGGATGCCGGTCAAGA-3'	291 – 274
P2Y_14_	Forward	5'-TTCCTTTCAAGATCCTTGGTGACT-3'	433 – 456
NM_014879	Reverse	5'-GCAGAGACCCTGCACACAAA-3'	505 – 486
GAPDH	Forward	5'-CCACCCATGGCAAATTCC-3'	227 – 244
NM_002046	Reverse	5'-TGGGATTTCCATTGATGACAAG-3'	295 – 274

### Western blotting

Cells were lysed in RIPA buffer containing 150 mM NaCl, 50 mM Tris-Cl (pH 7.2), 1% sodium deoxycholate, 0.1% SDS, 1 μg/mL aprotinin, 1 mM EGTA, 1 mM PMSF, and 1 mM sodium orthovanadate. After incubation in ice for 20 minutes on a shaking platform, the samples were centrifuged at 10,000 × g for 5 minutes at 4°C. Proteins were mixed with the sample buffer (50 mM Tris-Cl, pH 6.8, 10% glycerol, 2% SDS, 1% mercaptoethanol, and 0.1% bromophenol blue), heated to 95°C for 5 minutes, and separated on a 10% SDS-PAGE gel. The gel was transferred to polyvinylidene difluoride membranes (Amersham Pharmacia Biotech, Buckingshire, UK) and blocked in TBST (20 mM Tris-Cl, pH 7.6, 137 mM NaCl, 2.7 mM KCl, and 0.1% Tween 20) containing 5% (v/v) nonfat milk powder for 2 hours at room temperature. The membrane was incubated with rabbit polyclonal antibodies (Alomone Labs, Jerusalem, Israel) against P2X_1 _receptor, P2X_4 _receptor, P2X_7 _receptor, P2Y_1 _receptor, or P2Y_11 _receptor in TBST for 2 hours at room temperature, washed with TBST, and incubated with secondary anti-rabbit IgG (Amersham Pharmacia Biotech) in TBST for 1 hour. After the membrane was washed in TBST, protein bands were visualized using Western Lightning (PerkinElmer Life Sciences Inc., Gaithersburg, MD, USA). To compare protein loading, the blot was re-probed with anti-GAPDH antibody (Novus Biologicals, Littleton, CO, USA).

### Intracellular Ca^2+ ^measurements

The [Ca^2+^]_i _was measured using a single-cell microscopy technique with Fura 2 [31,37]. B cells and LCLs were suspended in culture and allowed to attach to glass coverslips coated with Poly-_L_-lysine (100 μg/mL; Sigma-Aldrich, St. Louis, MO, USA) and incubated for at least 3 hours before use. The cells were loaded with the cell-permeable Ca^2+ ^indicator Fura 2-AM (5.0 μM; Molecular Probes, Eugene, OR, USA) in the culture medium for 1 hour at room temperature and then washed and bathed in an external solution (135 mM NaCl, 5 mM KCl, 2 mM CaCl_2_, 1 mM MgCl_2_, 10 mM glucose, and 10 mM HEPES at pH 7.4) for at least 20 minutes before Ca^2+ ^measurements were made. Glass coverslips were placed into a chamber (Warner Instrument, Hamden, CT, USA) on an inverted microscope (Olympus, Tokyo, Japan), and the fluorescence intensities of the Fura-2-loaded cells were measured using a digital fluorescence imaging system. Discrete bandwidth excitation light (340 nm, 380 nm) was delivered to the epifluorescence attachment of the microscope through a quartz fiber-optic guide. The fluorescence emitted by the Fura-2-loaded cells was passed through a 510-nm-long pass filter, and images were obtained using a cooled charge-coupled device camera (Roper Scientific, Trenton, NJ, USA). Fluorescent video images were averaged, digitized (0.3–1.0 Hz), and analyzed using Metafluor acquisition and analysis software (Universal Imaging Corp, West Chester, PA, USA). Individual cells in the field of view were selected and paired 340/380 images were subtracted from the background. The Fura-2 fluorescence ratios, indicative of changes in [Ca^2+^]_i_, were calculated and their changes were extracted over time. All experiments were performed at room temperature, and the external solution and drugs were perfused at a rate of 2 mL/min by gravity. Data were expressed as the ratio of fluorescence due to excitation at 340 nm and at 380 nm (F340:380). In some experiments, a nominally Ca^2+^-free medium was used, which was identical in composition, except for the omission of CaCl_2_.

### Statistics

Data are presented as the mean ± SEM, and *n *indicates the number of independent experiments or the number of cells used to measure [Ca^2+^]_i_. Statistical significance was determined using one-way ANOVA or Student's *t *test; *p *< 0.05 was considered significant.

## Authors' contributions

DHL collected and analyzed the data and wrote the paper.

KSP supervised Ca^2+ ^measurement procedures and participated in writing the manuscript.

IDK originated the idea for the research and revised the manuscript.

JWK collected the data and participated in writing the manuscript.

BGH oversaw the collection and analysis of data and revised the manuscript.

All authors read and approved the final manuscript.
